# Screening and brief interventions with violently injured young trauma patients

**DOI:** 10.1186/1940-0640-8-S1-A78

**Published:** 2013-09-04

**Authors:** Laura J Veach, Regina R Moro

**Affiliations:** 1 Department of Counseling, University of North Carolina Charlotte, Charlotte, NC, and the Department of Surgery, Wake Forest School of Medicine, Winston Salem, NC, USA; 2 Department of Counseling, Barry University, Miami, FL, USA

## 

One key area of current prevention is influenced by alarming statistics about youth, alcohol and violence: 16 young people are murdered and 112 are violently injured daily in the US. Leading researchers recognize that alcohol and violence are major factors in the leading causes of death among youth. Trauma centers are key locations for services aimed at reducing future violent incidents (recidivism and retaliation) and alcohol misuse through the implementation of brief interventions. Multiple beneficial outcomes associated with culturally competent counseling interventions, including lower violent crime arrests after trauma center discharge and lower health care costs are anticipated yet unknown. Our objective was to examine the feasibility of providing alcohol screening in combination with violence screening and 2 brief counseling intervention (AVSBI) sessions to hospitalized trauma patients (15-25 years) with violence-related injuries in two Level I Trauma Centers in the US. Following consent protocol, screening tools were administered to assess violence risk factors, overall functioning and risky drinking with trauma patients (ages 15-25) with severe violence-related injuries followed by two brief interventions. Feasibility was assessed at both hospital trauma centers. Preliminary results pertaining to barriers and receptivity to AVSBI indicate overall positive receptivity and low decline rates; barriers were primarily institution-related in both hospital trauma centers. Trends noted in patient interactions were analyzed in a qualitative manner determining pertinent feasibility themes. AVSBI yielded positive trends when addressing both alcohol misuse and violence risk factors in violently-injured hospitalized youth. Further large scale study is warranted.

